# Re-emergence of circulating non-malignant B cells as a prognostic biomarker in chronic lymphocytic leukaemia

**DOI:** 10.1038/s41598-025-16558-5

**Published:** 2025-09-26

**Authors:** Yeong Jer Lim, Andrew Duckworth, Kim Clarke, Melanie Oates, Indrani Karpha, Matthew Gornall, Nagesh Kalakonda, Joseph Slupsky, Andrew Pettitt

**Affiliations:** 1 Department of Molecular & Clinical Cancer Medicine, University of Liverpool, Ashton St, Liverpool, L69 3GE UK; 2 The Clatterbridge Cancer Centre NHS Foundation Trust, Liverpool, UK; 3 Liverpool Clinical Trials Centre, University of Liverpool, Liverpool, UK

**Keywords:** Cancer, Oncology

## Abstract

To explore the relevance of non-malignant B cells (NMBCs) in chronic lymphocytic leukaemia (CLL), 201 blood samples from 79 previously untreated CLL patients receiving chemoimmunotherapy (CIT) in a phase III clinical trial were subjected to mass cytometry analysis using a bespoke panel of 24 antibodies. One memory (CD27^+^CD38^−^) and two naïve (CD27^−^CD38^+^) CD19^+^ clusters with a non-malignant phenotype (NMP; CD5^−^CD43^−^ROR1^−^CD20^hi^CD79b^hi^CD81^+^) were identified by FlowSOM and UMAP algorithms. NMP clusters were most prevalent in healthy controls, almost undetectable in untreated and relapsed CLL, and variable following CIT, where they correlated with haematopoietic recovery (naïve NMP clusters) and polyclonal antibody production (memory NMP cluster). Larger NMP cluster size at the early post-treatment timepoint was associated with a significantly longer progression free survival (PFS; HR 0.40 [95% CI: 0.22–0.75]; *P* = 0.003). Similar findings were obtained when NMBCs were prospectively defined by Boolean gating using the non-redundant features of the NMP clusters. NMBCs measured in this way negatively correlated with MRD and provided complementary prognostic information in patients with persistent MRD. Our findings raise the possibility that re-emergence of circulating NMBCs after treatment reflects clearance of CLL from secondary lymphoid organs, thereby complementing MRD as a biomarker of bone marrow clearance.

**Supplementary Information:**

The online version contains supplementary material available at 10.1038/s41598-025-16558-5.

## Introduction

Chronic lymphocytic leukaemia (CLL) is characterised by the clonal expansion of CD5^+^ B lymphocytes, which accumulate in the blood, bone marrow, and secondary lymphoid organs. A dynamic interplay exists at sites of tissue involvement whereby CLL cells shape the immune microenvironment and perturb normal immune function while at the same time relying on non-malignant immune cells for survival and proliferation^1–3^. An increasing body of literature has implicated non-malignant B cells (NMBCs) as active participants, rather than passive bystanders, within the cancer microenvironment^4,5^. Despite this, their role in CLL has received little attention, possibly owing to their relative paucity compared to the malignant CLL cells and the difficulty of reliably identifying them. The advent of high-dimensional single-cell technologies, coupled with the highly distinctive immunophenotypic features of CLL cells^6^, provides an opportunity to address this knowledge gap through a subtractive approach.

Here, we report the findings of a high-dimensional mass cytometry (CyTOF) analysis of circulating B cells in a highly annotated, well-defined cohort of CLL patients enrolled into the phase III RIAltO trial which compared alternative frontline chemoimmunotherapy (CIT) regimens. Using this approach, we were able to reliably quantify circulating NMBCs before and after treatment, as well as at disease progression, and relate these measurements to multiple baseline characteristics and clinical outcomes including progression free survival (PFS), overall survival (OS), attainment of measurable residual disease (MRD) negativity, and post-treatment cytopenias and hypogammaglobulinaemia. In doing so, we have shown for the first time that recovery of circulating NMBCs in CLL provides prognostic information complementary to MRD assessment and may be linked to restoration of haematopoietic function and polyclonal antibody production.

## Materials and methods

### Patient selection

All patients were enrolled in the phase III randomised controlled RIAltO trial (NCT01678430; EudraCT 2011-000919-22; Research Ethics Committee (REC) reference 11/NW/0548)^(7)^ between September 2014 and March 2016 and received at least 12 weeks of CIT, comprising ofatumumab combined with either bendamustine or chlorambucil, together with either idelalisib or placebo. A detailed description of the RIAltO trial is available in Supplementary Information. Patients were excluded if suitable samples were not available. Further details of patient selection are provided in the CONSORT diagram in Figure S1. REC approval was obtained for the collection and analysis of CLL patients (REC reference 19/NW/0573) and healthy controls (REC reference 16/NW/0810). The study was performed in accordance with the Declaration of Helsinki and relevant ethical guidelines for research in humans, with written informed consent obtained from all patients.

### Clinical and laboratory data

Clinical and laboratory data were collected as part of the RIAltO trial. Pre-treatment information included age, sex, stage, performance status, recurrent chromosomal abnormalities, IGHV mutation status, and treatment received. Chromosomal abnormalities were detected using the Vysis CLL FISH probe kit (Abbott, UK). The cut-off thresholds used to define the presence of specific abnormalities were as follows: ≥7% for 17p deletion, ≥ 6% for 11q deletion, ≥ 5.5% for monoallelic and ≥ 1.5% for biallelic 13q deletion, and ≥ 2.5% for trisomy 12. Longitudinal data included full blood count and serum immunoglobulin levels measured at a timepoint closest to the CyTOF analysis. The median time between CyTOF analysis and these laboratory measurements was 0 (interquartile range (IQR): 0–2) days for blood counts and 1 (IQR: 0–58) day for immunoglobulin levels. Follow-up data was available to April 2021. Endpoints included PFS, OS and attainment of MRD negativity in the bone marrow which was measured by flow cytometry 8–12 weeks after completing CIT in accordance with the European Research Initiative on CLL (ERIC) recommendations for MRD detection^6,7^. All tests were performed in medical laboratories accredited by the United Kingdom Accreditation Service (UKAS) against the international standard ISO 15,189.

### Sample Preparation

Blood samples from CLL patients were collected in EDTA and transported within 24 h to the UK CLL Biobank (REC 14/NW/1014). Healthy control samples consisted of donor buffy coats obtained from the Speke blood bank and stored in the Liverpool Blood Disease Biobank (REC 16/NW/0810). Peripheral blood mononuclear cells (PBMCs) from all samples were isolated by centrifugation over Lymphoprep (STEMCELL technologies, Ca) and cryopreserved in 10% DMSO at −80 °C. Prior to analysis, samples were thawed at 37 °C, diluted incrementally in ice-cold cell culture media comprising Roswell Park Memorial Institute (RPMI)−1640 and 10% fetal bovine serum (FBS), and washed twice before counting. Two reference control samples, comprising biological replicates of a CLL patient and a healthy control, were also included in each batch to allow the assessment of batch-specific effects.

### Live metal-tagged barcoding

Individual blood mononuclear cell samples were stained for 30 min at 4 °C with pre-allocated, 8-choose-2 combinations of 89Yb-, 106Cd-, 110Cd-, 111Cd-, 113Cd-, 114Cd-, 115In-, and 116Cd-CD45 (HI30, Biolegend) antibodies. Barcoded samples were then washed (550xg/4°C/5 minutes) twice with Maxpar Cell Staining Buffer (CSB; Standard Biotools^™^, US) and pooled together to create a single, multiplexed sample^8^.

### Mass cytometry (CyTOF)

Pooled samples were incubated with a surface antibody cocktail (Table 1) diluted to their previously optimised concentrations. After an incubation period of 45 min on ice, cells were washed twice with CSB and Maxpar phosphate buffered saline (PBS; Standard Biotools^™^, US) and then stained for viability by resuspending in a PBS-cisplatin solution (prepared by diluting Cell-ID™ cisplatin-195Pt (Standard Biotools^™^, US) with PBS at a ratio of 1:1000) for 5 min at room temperature. Cisplatin quenching was then performed by adding RPMI supplemented by 20% FBS at 5 times the total PBS-cisplatin volume used. The cells were then fixed with 1.6% formaldehyde (ThermoFisher Scientific, UK), left overnight at 4 °C, washed, and resuspended in intercalation solution comprising 125nM Maxpar^®^ Intercalator-Ir and Maxpar^®^ Fix and Perm Buffer (Standard Biotools^™^, US) at a 1:2000 ratio. After incubation for 60 min, cells were washed with Maxpar^®^ Cell Acquisition Solution (Standard Biotools^™^, US), and EQ calibration beads were added at a 1:10 ratio. Finally, samples were loaded into the Helios instrument (Standard Biotools^™^, US), where cell events were recorded at a rate of 200–400 cells/second.

**Table 1:** Surface antibody panel used for cytof analysis. IH: in-house conjugation, P: purchased pre-conjugated.

Marker	Clone	Isotope	Titration	Source (Conjugation method)
CD19	HIB19	142Nd	1:50	Standard Biotools, UK (P)
CD14	RMO52	148Nd	1:200	Standard Biotools, UK (P)
CD3	UCHT1	170Er	1:200	Standard Biotools, UK (P)
CD16	3G8	209Bi	1:200	Standard Biotools, UK (P)
HLADR	L243	174Yb	1:100	Standard Biotools, UK (P)
CD5	UCHT2	143Nd	1:50	Standard Biotools, UK (P)
CD81	5A6	145Nd	1:100	Standard Biotools, UK (P)
CD20	2H7	147Sm	1:100	Standard Biotools, UK (P)
CD79b	3A2-2E7	150Dy	1:100	ThermoFisher, UK (IH)
ROR1	2A2	155Gd	1:50	Biolegend, UK (IH)
CD43	1G10	168Er	1:400	ThermoFisher, UK (IH)
CD38	HIT2	144Nd	1:100	Standard Biotools, UK (P)
IgD	IA6-2	146Nd	1:100	Standard Biotools, UK (P)
CD24	ML5	152Sm	1:100	Biolegend, UK (IH)
CD27	L128	158Gd	1:100	Standard Biotools, UK (P)
sIgM	MHM-88	176Yb	1:50	Biolegend, UK (IH)
CD138	MI15	166Er	1:100	Biolegend, UK (IH)
CD49d	9F10	141Pr	1:100	Standard Biotools, UK (P)
CD84	CD84.1.21	154Sm	1:100	Standard Biotools, UK (P)
CD180	RP105	169Tm	1:100	Biolegend, UK (IH)
CCR7	G043H7	167Er	1:100	Standard Biotools, UK (P)
CXCR4	12G5	175Lu	1:100	Standard Biotools, UK (P)
CXCR5	RF8B2	164Dy	1:100	Standard Biotools, UK (P)
CD62L	DREG-56	153Eu	1:100	Standard Biotools, UK (P)

### Cleaning and analysis of cytof dataset

Raw data was first normalised against EQ beads using the CyTOF Software v8.0 and analysed using the online Cytobank software (Standard Biotools^™^, US). Doublets were removed by the gating of Intercalator-Ir and gaussian parameters, while viable cells were identified by gating 195^Pt^ negative events. Events were then debarcoded by implementing a single-cell debarcoding algorithm^9^ which assigns each cell to its respective sample based on the expression profile of barcoding antibodies, using R packages *FlowCore* and *CATALYST*^10^. Analysis of reference control samples included in every batch showed similar marker expression profiles, negating the need for batch correction (Figure S2). Where possible, all evaluable cells from each sample were analysed, with a median of 52,456 cells analysed per sample (IQR: 19,039–87,117), ensuring robust sensitivity for detecting NMBCs across most samples. Unsupervised analysis involving algorithms such as Uniform Manifold Approximation and Projection (UMAP) and FlowSOM was performed using the *CATALYST* package, based on the expression profiles of all markers listed in Table 1 and using a randomly generated seed. FlowSOM clustering was performed using a 15 × 15 self-organizing map (SOM) grid, followed by hierarchical consensus clustering to define 50 metaclusters. UMAP analysis was conducted with 15 nearest neighbours and a minimum distance of 0.01. Supervised analysis using Boolean gating was performed using the Cytobank software. The frequencies of FlowSOM metaclusters and gated B cell populations were calculated and reported as proportions of total mononuclear cells in each sample.

### Statistical analysis of clinical correlations

All statistical analyses were conducted using R software (version 4.0.2). The Mann-Whitney U test was used to compare NMBC proportions across different patient groups. Spearman’s rank correlation coefficient was employed to assess the correlation between NMBCs and other variables. Uni- and multivariable analyses for censored outcomes were performed using Cox proportional hazard regression in the *survival* R package, while PFS and OS between patient groups were compared using the Kaplan-Meier method and log-rank test. The optimal cut-off point for NMBC proportions in predicting outcomes, as well as their complementarity with other covariates, was explored using recursive partitioning analysis (RPA) via the classification and regression tree (CART) method implemented in the *partykit* package^11^. A p value of 0.05 was used throughout to determine statistical significance.

## Results

### Patient and sample characteristics

Overall, 201 samples from 79 patients with CLL and three healthy controls (HCs) were analysed by mass cytometry (Table 2). All patients were treatment-naïve and considered unfit for fludarabine, cyclophosphamide and rituximab (FCR). Sixty-two of the 79 patients (78%) were male and the median age was 75 years [IQR 70.5–79]. IGHV gene segments were > 2% mutated in 26 patients (33%), while 12 patients (15%) had unfavourable chromosomal abnormalities (17p- or 11q-) based on the Döhner hierarchical classification^12^. Forty-one (52%) and 38 (48%) patients received bendamustine or chlorambucil, respectively, while 35 (44%) and 44 (56%) received additional idelalisib or placebo, respectively. Samples were collected before treatment (*n* = 79), early after CIT (median 11.2 [IQR 11–11.7] months; *n* = 59), late after CIT (18.5 [16.1–22.6] months; *n* = 39), and at disease progression (*n* = 20).

**Table 2:** Baseline characteristics of all included patients. ECOG: Eastern cooperative oncology group. Chromosomal abnormalities were categorised according to the Döhner hierarchical classification.

Variable	No. of patients (%)	
n	79	
Median age [IQR] (years)	75 [70.5–79]	
Sex	Male	62 (78%)
	Female	17 (22%)
ECOG performance score	0–1	71 (90%)
	2–3	8 (10%)
Binet staging	A	12 (15%)
	B	25 (32%)
	C	42 (53%)
Chromosomal abnormalities	17p deletion	1 (1%)
	11p deletion	11 (14%)
	Trisomy 12	16 (20%)
	13q deletion	29 (37%)
	No aberrations	20 (25%)
	Unknown	2 (3%)
IGHV status	Mutated	26 (33%)
	Unmutated	34 (43%)
	Unknown	19 (24%)
Chemotherapy allocation	Bendamustine	41 (52%)
	Chlorambucil	38 (48%)
Allocation to idelalisib vs. placebo	Idelalisib	35 (44%)
	Placebo	44 (56%)

### Identification of non-malignant B cells

**Fig. 1:**
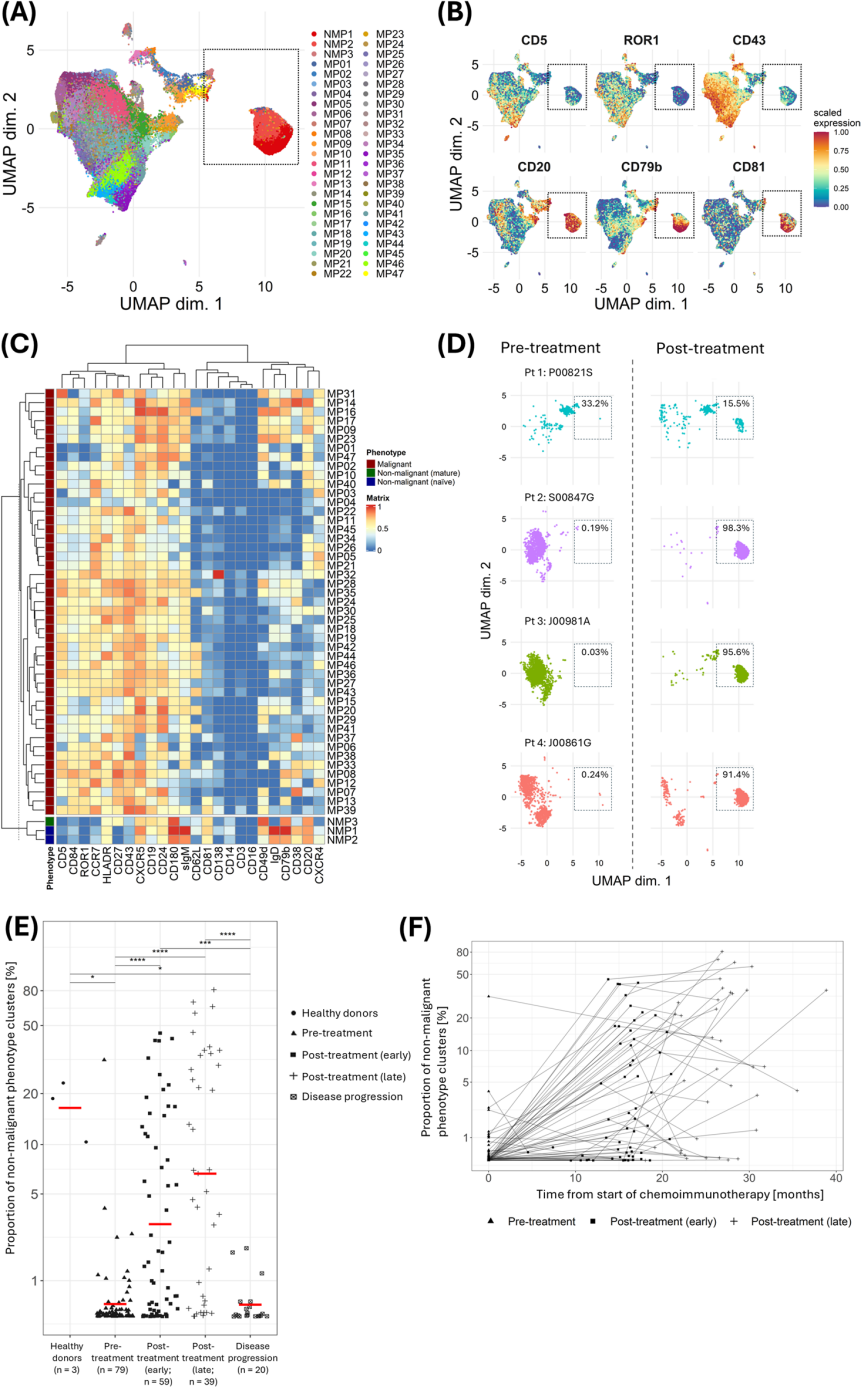
Detection and quantification of non-malignant B cells. **(A)** UMAP of CD19^+^ B cells from all analysed samples, annotated by their corresponding FlowSOM clusters. **(B)** UMAPs showing expression of established chronic lymphocytic leukaemia (CLL) markers. **(C)** Heatmap of CD19^+^ clusters identified by FlowSOM. **(D)** UMAPs of representative paired samples at pre- (left) and post-treatment (right) timepoints, illustrating the heterogeneity of MP cells and homogeneity of NMP cells between individual CLL patients. Gates and proportion of NMP clusters within CD19^+^ B cells were added. **(E)** Relative size of NMP and MP clusters within the overall mononuclear cell population in healthy controls and CLL patients at pre-treatment and post-treatment timepoints*. **(F)** Relative size of NMP clusters within the overall mononuclear cell population in all analysed patient samples plotted against the time of measurement after the start of chemoimmunotherapy*. Datapoints are joined where they relate to the same patient. MP: malignant phenotype, NMP: non-malignant phenotype. *Log10 transformation was applied to the y-axis to aid visualisation.

To distinguish CLL cells from NMBCs, CD19^+^ cells from all samples underwent pooled analysed using dimensionality reduction (UMAP) and unsupervised clustering (FlowSOM) algorithms. The aggregated UMAP representing CD19^+^ cells from all samples revealed two distinct clusters along the UMAP1 axis, differentiated by the expression of CLL-specific markers including CD5, CD20, CD79b, CD43, ROR1, and CD81 (Fig. 1A & B; Figure S3). The larger cluster exhibited a marker profile typical of CLL cells (CD5^+^CD43^+^ROR1^+^CD20^lo/−^CD79b^lo/−^CD81^lo/−^) and was assumed to have a malignant phenotype (MP). In contrast, the smaller cluster displayed the converse marker expression pattern (CD5^−^CD43^−^ROR1^−^CD20^hi^CD79b^hi^CD81^+^) and was assumed to have a non-malignant phenotype (NMP). Using the FlowSOM algorithm, up to 50 different clusters were identified. Three of these clusters expressed NMP markers (Fig. 1C) and mapped onto the NMP B cells identified on UMAP. NMP B cells had mostly uniform marker profiles and could be categorised into memory or naïve B cells based on CD27, CD38, and surface immunoglobulin (Ig) expression^13^. In contrast, MP B cells displayed significant heterogeneity with variable expression patterns found in almost all B cell markers (Fig. 1D).

### Quantification of non-malignant B cells

NMP clusters, measured as a proportion of all mononuclear cells, were prevalent in HCs, infrequent in patients with untreated and relapsed CLL, and present in variable numbers following completion of CIT (Fig. 1E). No association was observed between the frequency of NMP clusters prior to and following CIT (Figure S4). The most abundant NMP clusters in HC and post-CIT CLL samples were those with a naïve B cell phenotype (CD27^−^CD38^+^), whereas the cluster with a memory B cell phenotype (CD27^+^CD38^−^) was the least abundant (Figure S5). Collectively, NMP clusters peaked at ~ 15–20 months after completion of CIT, after which they were sustained in some patients and declined in others (Fig. 1F). NMP clusters were higher in patients who had received bendamustine compared to chlorambucil, and in those who had received idelalisib compared to placebo, with statistically significant differences observed at the late post-CIT timepoint (Figure S6).

### Correlation between re-emergence of non-malignant B cells and Haematopoietic recovery

We next explored the biological and clinical significance of NMBC populations by relating them to key elements of the full blood count measured at a similar time period (Table 3; Figure S7). At the early post-CIT timepoint, a weak correlation was seen between the two immunologically naïve (CD27^−^CD38^+^) NMP clusters and haemoglobin concentration (Spearman’s Rho correlation (r_s_): 0.36, *P* = 0.005), neutrophil count (r_s_: 0.37, *P* = 0.004) and platelet count (r_s_: 0.32, *P* = 0.014). A correlation was also seen between the memory (CD27^+^CD38^−^) NMP cluster and Hb concentration at the early timepoint (r_s_: 0.29, *P* = 0.03), and between the platelet count and both naïve (r_s_: 0.41, *P* = 0.01) and memory (r_s_: 0.40, *P* = 0.01) NMP clusters at the later post-CIT timepoint. Together, these observations demonstrate a possible link between re-emergence of NMBCs and recovery of haemopoietic function.

**Table 3:** Correlation between re-emergence of non-malignant B cells and recovery of blood counts and polyclonal antibody production after treatment. Table depicting spearman’s correlation coefficients between non-malignant clusters with a Naïve or memory phenotype, measured at early or late post-treatment timepoints, and components of the full blood count and serum Immunoglobulin levels. Statistically significant correlations are shown in bold. *P < 0.05, **P < 0.01, ***P < 0.001. $^{\dagger}$At the early post-treatment timepoint, one patient was excluded due to missing serum Immunoglobulin data, while an additional patient had available data for IgM and IgA but lacked information on IgG levels. One patient was excluded at the late post-treatment timepoint for the same reason.

Post-treatment timepoint		Early (*n* = 59)		Late (*n* = 39)	
Non-malignant B clusters		CD27^-^CD38^+^ (naïve)	CD27^+^CD38^-^(memory)	CD27^-^CD38^+^ (naïve)	CD27^+^CD38^-^(memory)
Full blood count	Hb	0.36**	0.29*	0.17	0.26
	Neutrophil count	0.37**	0.17	0.26	0.06
	Platelet count	0.32*	0.12	0.41*	0.40*
Serum immunoglobulin concentration$^{\dagger}$	IgA	0.14	0.40**	0.08	0.33*
	IgM	−0.06	0.14	0.33*	0.43**
	IgG	0.02	0.37**	0.15	0.33*

### Correlation between re-emergence of non-malignant B cells and polyclonal antibody production

We also related the re-emergence of NMBCs to serum immunoglobulin levels at the time of NMBC measurement. At the late post-CIT timepoint, a weak to moderate correlation was observed between the memory (CD27^+^CD38^−^) NMP cluster and serum concentrations of IgA (r_s_: 0.33, *P* = 0.040), IgM (r_s_: 0.43, *P* = 0.007), and IgG (r_s_: 0.33, *P* = 0.046; Table 3; Figure S8). A correlation was also seen between the naïve (CD27^−^CD38^+^) NMP clusters and IgM levels (r_s_: 0.33, *P* = 0.044) at the late post-CIT timepoint, and between the memory NMP cluster and levels of IgA (r_s_: 0.40, *P* = 0.002) and IgG (r_s_: 0.37, *P* = 0.005) at the early post-CIT timepoint. Together, these findings demonstrate a possible link between the re-emergence of NMBCs and polyclonal antibody production.

### Re-emergence of non-malignant B cells is an independent prognostic factor

**Fig. 2:**
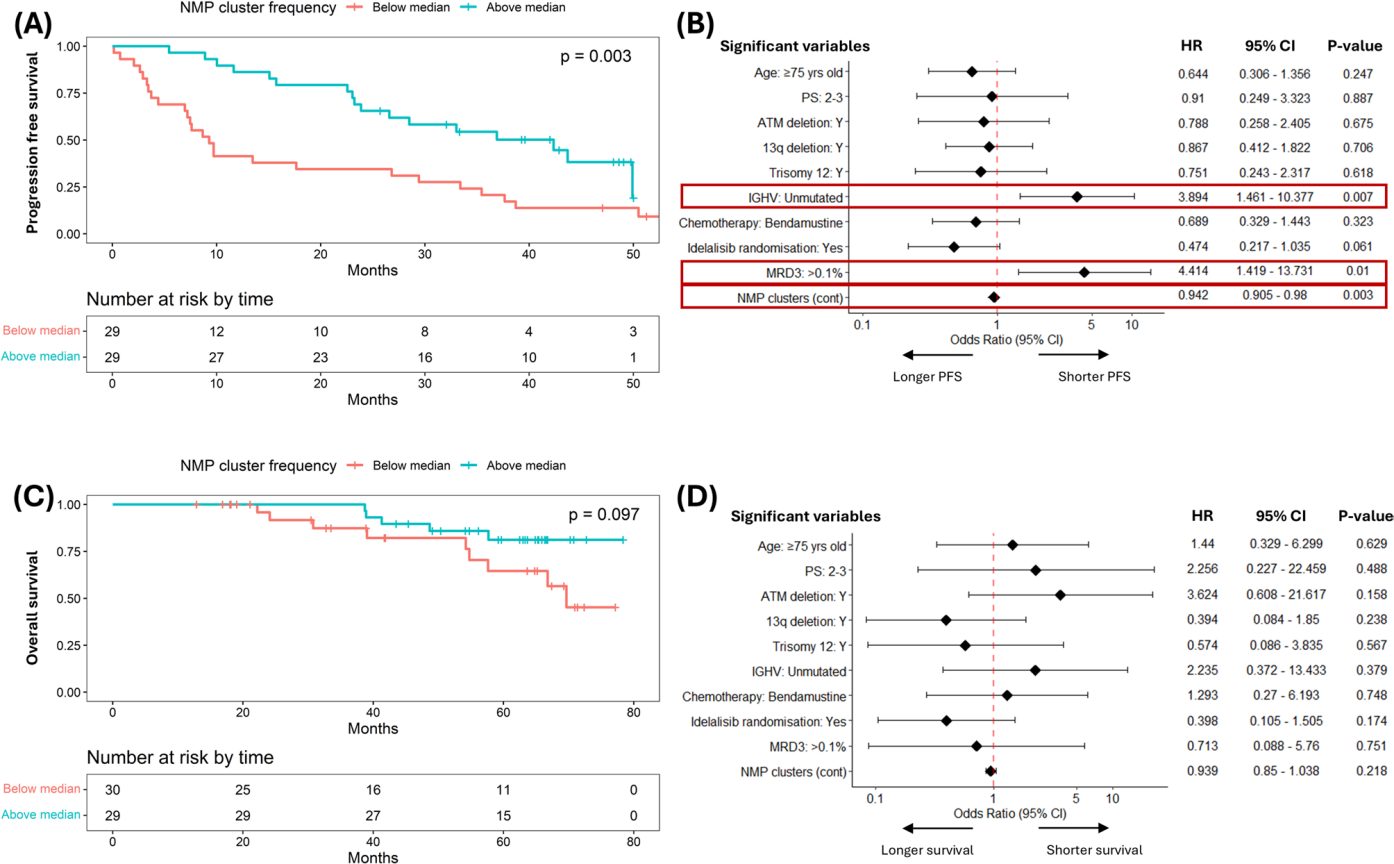
Prognostic effect of non-malignant B-cell clusters. **(A)** Kaplan-Meier (K-M) plot and log-rank test of progression free survival (PFS) comparing patients with FlowSOM-defined NMP clusters above or below the median value (1.52%) at the early post-CIT timepoint*. **(B)** Multivariable Cox proportional hazard analysis assessing the association between PFS and baseline characteristics, treatment allocation, post-chemoimmunotherapy (CIT) MRD status (using a cut-off of 10^−3^ since only five patients achieved 10^−4^), and NMP cluster size at the early post-CIT timepoint*. **(C)** K-M plot and log-rank test of overall survival (OS) comparing patients with NMP clusters above or below the median (1.52%) at the early post-CIT timepoint. **(D)** Multivariable Cox proportional hazard analysis assessing the association between OS and baseline characteristics, treatment allocation, post-CIT MRD status (using a cut-off of 10^−3^), and NMP cluster size at the early post-CIT timepoint. NMP cluster frequency was analysed as a continuous variable in **(B)** and **(D)**. *One patient progressed prior to the post-treatment blood sample and was excluded.

Next, we explored the relationship between NMBC numbers at the early post-CIT timepoint and long-term disease outcomes measured from the same timepoint. Stratification based on the median NMP cluster size showed that patients with larger NMP clusters experienced significantly longer progression-free survival (PFS; median 42.4 [95% CI: 26.6–NR] months vs. 9.3 [7.2–29.4] months; HR: 0.40 [0.22–0.75], *P* = 0.003; Fig. 2A). Consistent with this finding, univariable cox proportional hazard (coxph) analysis identified the addition of idelalisib, as well as post-treatment measurements of haemoglobin, MRD and the NMP cluster size, as factors associated with PFS. All of these factors, with the exception of idelalisib, remained significant in the multivariable analysis (Table 4). A similar trend was also observed at the late post-CIT timepoint (Figure S9). However, 12 of the 15 (80%) evaluable patients with elevated NMBC levels at this later timepoint were also identified as those who had more NMBCs at the early post-CIT timepoint, suggesting that the elevated NMBCs at this timepoint reflect a sustained recovery that mostly occurred earlier, rather than the emergence of a distinct subgroup.

**Table 4:** Univariable and multivariable analyses for progression-free survival following completion of chemoimmunotherapy, with estimated effect (Est), 95% confidence interval (CI) and statistical significance for all potential covariates. ECOG: Eastern cooperative oncology group, NMP: non-malignant phenotype. *Samples with no available information were excluded.

Covariate(s)	Level(s)	Univariable analysis		Multivariable analysis	
		Est (95% CI)	*P*-value	Est (95% CI)	*P*-value
Age (years)	<75	Reference			
	≥75	0.735 (0.397, 1.358)	0.325		
ECOG performance score	0–1	Reference			
	2–3	0.752 (0.266, 2.124)	0.591		
Sex	Female	Reference			
	Male	1.127 (0.539, 2.36)	0.75		
Binet stage	A/B	Reference			
	C	1.022 (0.558, 1.872)	0.944		
IgHV mutational status	Mutated	Reference			
	Unmutated	1.691 (0.834, 3.426)	0.145		
11p deletion	No	Reference			
	Yes	2.045 (0.889, 4.704)	0.092		
13q deletion	No	Reference			
	Yes	1.151 (0.622, 2.127)	0.654		
Trisomy 12	No	Reference			
	Yes	0.728 (0.335, 1.581)	0.422		
Chemotherapy received	Chlorambucil	Reference			
	Bendamustine	0.604 (0.326, 1.122)	0.111		
Idelalisib randomisation	Placebo	Reference		Reference	
	Idelalisib	0.427 (0.223, 0.817)	0.01	0.630 (0.321, 1.238)	0.18
Post-treatment FBC:					
Haemoglobin (g/dL)	≥12	Reference		Reference	
	<12	3.211 (1.678, 6.143)	0.0004	3.484 (1.649, 7.364)	0.001
Neutrophil count (x10^9/L)	≥1.5	Reference			
	<1.5	1.255 (0.297, 5.297)	0.757		
Platelet count (x10^9/L)	≥150	Reference			
	<150	1.566 (0.856, 2.865)	0.146		
Post-treatment immunoglobulin levels:					
Serum IgG (g/L)*	Continuous	1.036 (0.936, 1.146)	0.498		
Serum IgA (g/L)*	Continuous	0.97 (0.785, 1.199)	0.78		
Serum IgM (g/L)*	Continuous	0.9 (0.684, 1.185)	0.454		
Post-treatment MRD	<0.1%	Reference		Reference	
	≥0.1%	3.63 (1.527, 8.628)	0.004	4.94 (1.858, 13.133)	0.001
NMP clusters	Continuous	0.953 (0.92, 0.987)	0.007	0.958 (0.924, 0.993)	0.019

Given the heterogeneity of the analysed CLL cohort, which included patients with variable CLL prognostic factors and different treatment allocations, we constructed an additional coxph regression model incorporating age, ECOG performance status, recurrent chromosomal abnormalities, IGHV mutation status, treatment allocation, end-of-treatment MRD and the post-treatment NMP cluster size. In this model, NMP cluster size emerged as one of only three independent predictors of PFS, the other two being unmutated IGHV and attainment of MRD3 negativity (Fig. 2B). Additionally, restricting our analysis to samples with at least 1,000 total cells analysed (*n* = 47) confirmed the persistent association between NMP cluster size and PFS among samples with assay sensitivity of at least 0.001% (i.e., 1 in 1,000 cells; Figure S10).

Although a trend toward longer survival was observed among patients with larger NMP cluster sizes (Fig. 2C), it did not reach statistical significance in univariable and multivariable analyses, likely owing to the small number of events and the rescuing effects of subsequent anti-CLL therapies (Fig. 2D, Table S1). Overall, these findings identify re-emergence of NMP clusters as an independent prognostic factor for predicting future risk of progression in CLL.

**Fig. 3:**
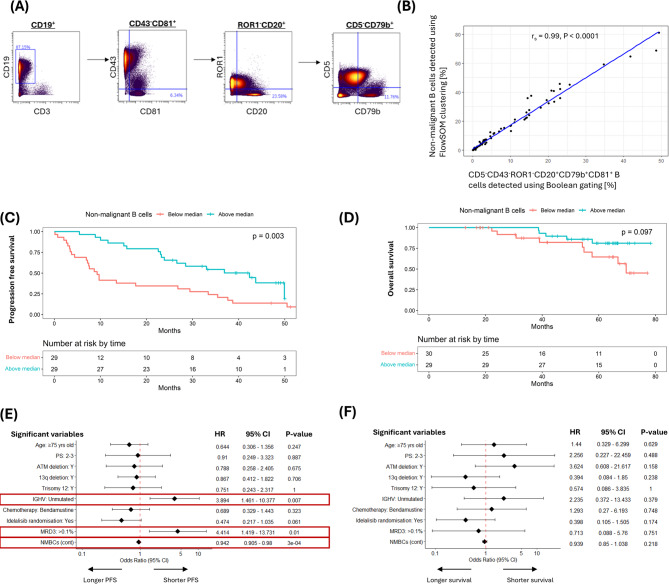
Prognostic effect of prospectively defined non-malignant B cells. **(A)** Boolean gating strategy used to identify CD5^−^CD43^−^ROR1^−^CD20^+^CD79b^+^CD81^+^ NMBC. **(B)** Scatter plot and linear regression analysis comparing NMBCs detected using a supervised (Boolean gating) vs. unsupervised (FlowSOM clustering) approach. **(C)** K-M plot and log-rank test of PFS* comparing patients with gated NMBC above or below the median value (0.77%) at the early post-treatment timepoint. **(D)** K-M plot and log-rank test of OS comparing patients with gated NMBC above or below the median value (0.77%) at the early post-treatment timepoint. **(E)** Multivariable Cox proportional hazard analysis assessing the association between PFS and baseline characteristics, treatment allocation, post-CIT MRD status (using a cut-off of 10^−3^ since only five patients achieved 10^−4^), and size of gated NMBC population at the early post-CIT timepoint.* **(F)** Multivariable Cox proportional hazard analysis assessing the association between OS and baseline characteristics, treatment allocation, post-CIT MRD status (using a cut-off of 10^−3^), and size of gated NMBC population at the early post-CIT timepoint. NMP cluster frequency was analysed as a continuous variable in **(E)** and **(F)**. *One patient relapsed prior to the post-treatment blood sample and was excluded from TTP analysis.

While the unsupervised FlowSOM approach is suitable for biomarker discovery, its data dependence makes it unsuitable for biomarker validation and clinical application. To address this limitation, supervised gating strategies were devised to prospectively identify NMBCs using a panel of markers that captured the key non-redundant features of NMP clusters (CD5^−^CD43^−^ROR1^−^CD20^+^CD79b^+^CD81^+^). When comparing NMBC frequencies detected by supervised gating vs. FlowSOM, correlation improved with additional NMP-defining markers. Regardless of the combination of NMBC-defining markers used, consistent and robust correlation were only observed when at least seven markers (three lineage-defining and four NMBC-defining) were included, reaching a maximum when all nine markers were used (three lineage-defining and all NMBC-defining (CD5/CD43/ROR1/CD20/CD79b/CD81) markers; r_s_= 0.99, *P* < 0.0001; Fig. 3A and B; Figure S11). Furthermore, stratifying patients based on the median size of the prospectively defined NMBC (CD5^−^CD43^−^ROR1^−^CD20^+^CD79b^+^CD81^+^) population confirmed that the group with more of these cells had a significantly longer PFS (median 42.4 [95% CI: 26.6–NR] months vs. 9.3 [7.2–29.4] months; HR: 0.40 [0.22–0.75], *P* = 0.003) and a trend towards longer OS (median NR vs. 69.7 [57.6–NR] months; HR: 0.40 [0.13–1.22], *P* = 0.097; Figs. 3C-F). Similar to NMP clusters, the association between NMBCs and PFS persisted in a multivariable coxph model after adjusting for age, performance status, chromosomal abnormalities, IGHV mutation status, treatment allocation, and end-of-treatment MRD (Fig. 3E).

### Non-malignant B cells provide complementary prognostic information in patients with residual disease

**Fig. 4:**
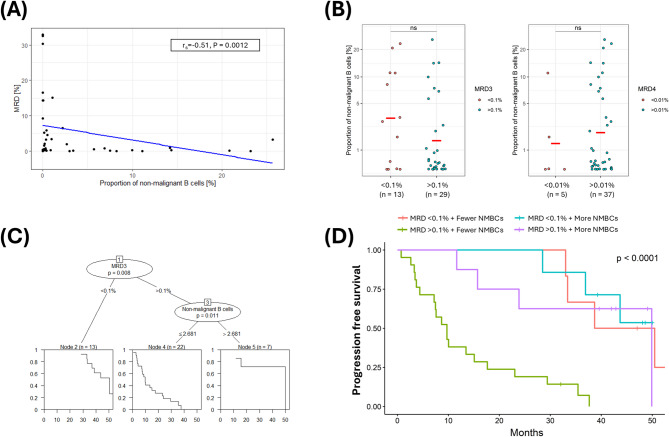
Correlation between prospectively defined non-malignant B cells and MRD, and their complementarity in predicting risk of progression following chemoimmunotherapy. **(A)** Scatter plot and linear regression analysis comparing MRD levels with prospectively defined NMBCs at early post-treatment timepoint among patients with available MRD results (*n* = 43). **(B)** Proportion of NMBC in patients with high or low MRD levels defined by cut-offs of 10^−3^ (left) or 10^−4^ (right). **(C)** Results of recursive partitioning analysis of the association between PFS and all available pre- and post-treatment covariates among patients with available MRD results (*n* = 43). **(D)** K-M plot and log-rank test of PFS among patients grouped by NMBC frequency using the RPA-defined threshold (2.68%) and their attainment of MRD3.

Given that the marker profile used to identify NMBCs was the reciprocal of that used to define CLL cells, we further examined the relationship between prospectively defined NMBCs (measured as a proportion of mononuclear cells in the blood) and MRD (measured as a proportion of CD45^+^ leukocytes in the bone marrow). While a negative correlation between these variables was observed at the early post-CIT timepoint (Fig. 4A), considerable heterogeneity in NMBC frequencies was evident regardless of MRD status (Fig. 4B). Furthermore, recursive partitioning analysis (RPA) of the association between progression-free survival and all available pre-treatment (age, sex, ECOG performance status, Binet stage, recurrent chromosomal abnormalities, IGHV mutation status, treatment allocation) and post-treatment (recent blood counts, immunoglobulin levels, MRD status, NMBC frequency) covariates revealed that only NMBC frequency, using a threshold of 2.68%, provided complementary prognostic information for patients with persistent MRD following CIT (Fig. 4C). Finally, stratification of patients by MRD status and NMBC frequency (based on the RPA-defined threshold) identified a subgroup with residual disease and lower NMBC counts who experienced notably shorter PFS (Fig. 4D).

## Discussion

In a field dominated by the investigation of malignant B cells, our study sheds new light on the relevance of NMBCs in in the context of frontline CLL therapy. Although other studies have investigated circulating NMBCs in this setting^14–18^, our findings are novel in that they describe previously unreported longitudinal changes, clinical associations, and prognostic effects. By applying an extensive panel of B cell markers coupled with an unsupervised approach to analysis, we were able to distinguish between CLL cells and NMBCs and further characterise the latter into mature and naïve subpopulations. Through serial measurement of NMBCs as a proportion of circulating mononuclear cells, we found that these cells are infrequent prior to therapy and exhibit variable recovery thereafter in a way that correlates with restoration of haematopoietic function and polyclonal antibody production. Crucially, we demonstrated that measurement of circulating NMBCs provides independent prognostic information that complements MRD assessment in predicting long-term disease outcomes.

There were several strengths to our study design. First, the utilisation of patient samples obtained from a prospective randomised controlled clinical trial ensured that the cohort of CLL patients was well defined, and that treatment allocation was evenly distributed across baseline variables. This approach minimised the impact of confounding variables associated with disease status, prior treatments, and other baseline characteristics. Embedding the study within a clinical trial also ensured that the clinical data with which the samples were annotated was reliable and complete, and that the samples themselves were collected, transported, processed, and stored in accordance with Good Clinical Practice, thereby optimising the quality and reliability of the laboratory data. Second, the simultaneous analysis of 24 surface markers at single-cell resolution created a high-dimensional dataset that not only allowed malignant and non-malignant B cells to be distinguished from one another but also enabled full visualisation of the NMBC architecture and derivation of clusters in an objective, unbiased way. Third, the use of Boolean gating to prospectively define NMBCs based on non-redundant features of MNP clusters enabled internal validation of the findings and established a set of parameters that could be readily employed in future studies.

The extremely low proportion of circulating NMBCs across all pre-treatment and progression samples is striking and may suggest a dilution effect resulting from the relative abundance of CLL cells in these settings. Alternatively, it could also reflect genuine variation in the absolute number of circulating NMBCs, which may be suppressed by CLL cells due to their infiltrative nature within primary and secondary lymphoid structures. Both mechanisms may account for the higher NMBC levels observed after bendamustine, which is more effective at cytoreduction than chlorambucil^19–21^, as well as the negative correlation between MRD and the re-emergence of NMBCs post-CIT. While further investigations is warranted, the variability in NMBC frequencies among patients with positive or negative MRD status suggests that the changes in NMBCs following treatment are not solely attributable to dilution by CLL cells but may also reflect the alleviation of CLL-associated suppression of normal lymphopoiesis.

In this context, the correlation between post-treatment NMBCs and serum immunoglobulin levels, albeit weak, is nevertheless relevant as it links circulating NMBCs with a robust indicator of B cell functionality. This, in turn, supports the idea that NMBCs recirculate between blood and secondary lymphoid organs and that their abundance in the blood therefore has a direct biological relevance. The fact that the correlation between NMBCs and serum immunoglobulin levels involved predominantly memory (CD27^+^CD38^−^) cells and was more pronounced at the late post-treatment timepoint suggests that polyclonal antibody production after treatment depends primarily on the expansion of memory B cells and their differentiation of into short-lived (IgM-producing) or long-lived (IgG- and IgA-producing) plasma cells over a sustained period of time.

Regarding the association between NMBCs and hematopoietic recovery, it is possible that NMBCs directly influence haematopoiesis, or vice versa^22^. However, it seems more likely that the two outcomes are separate manifestations of cellular regeneration at different sites of tissue involvement following clearance of the malignant clone. This being the case, the stronger correlation observed between NMBCs and haematopoietic recovery at the early post-treatment timepoint compared to the later timepoint likely reflects the divergent regeneration kinetics of different cell lineages at different anatomical sites.

To our knowledge, this study is the first to identify post-treatment circulating NMBCs as a prognostic factor in CLL, the association with PFS reaching a high level of statistical significance. Crucially, this association persisted after adjusting for a range of other variables known to influence outcome, including IGHV mutational status, treatment allocation and end-of-treatment MRD, thereby establishing NMBCs as an independent prognostic factor. Although post-treatment Hb levels were also associated with PFS in univariable analysis, the failure of the RPA model to identify Hb levels as an independent determinant of PFS suggests a dominant effect of NMBC. Given the negative correlation between NMBCs and MRD, the element of reciprocity involved in defining the two variables, and the established prognostic importance of MRD, it was important to understand how NMBCs added to the prognostic value of MRD. Notably, among patients with persistent MRD, the two variables offered complementary prognostic information by identifying a subgroup with both persistent MRD and fewer NMBCs (< 2.68% of overall mononuclear cell population), who experienced a significantly shorter PFS duration.

The biological basis for the prognostic effect of NMBCs and its complementarity with MRD is not clear, but potential explanations include a direct role of NMBCs in limiting CLL progression. Alternatively, and perhaps more plausibly, re-emergence of circulating NMBCs might be a marker of CLL clearance from secondary lymphoid organs such as lymph nodes and spleen, for which MRD might not be a reliable marker. Thus, although MRD levels in blood and bone marrow are highly concordant^7^, concordance with other anatomical sites is both unclear and difficult to predict given the distinct mechanisms involved in the homing of CLL cells to bone marrow vs. lymphatic tissues^23^ and the non-identical microenvironmental stimuli encountered by CLL cells at these different sites^24^. In keeping with this, it is well documented that lymph node enlargement can persist in patients who achieve low levels of MRD in the blood and bone marrow following treatment, and that such patients are more likely to experience early disease progression compared to those who achieve comparably low MRD levels in the context of a complete anatomical response^25^.

Measurement of NMBCs might therefore constitute a simpler, more reliable and less costly alternative to imaging as a way of assessing CLL clearance from secondary lymphoid organs, in which case combined measurement of MRD and NMBCs in a single blood test could provide a more comprehensive way of evaluating CLL clearance from all anatomical sites for the purposes of prognostication or informing on the optimal duration of therapy regimens that aim to achieve maximum cytoreduction^26,27^. Measurement of circulating NMBCs might also be a more informative way of assessing response to continuous single-agent therapy with BTK inhibitors, where low levels of MRD are rarely achieved and of limited value in predicting long-term disease outcomes^7,28,29^.

Our study had several limitations. First, due to the variable marker profile in CLL, NMP clusters were strictly defined as those with a marker profile that was the exact inverse of that typically associated with CLL^6^. While this approach increased confidence that the identified NMP clusters was confined to NMBCs, it also excluded non-malignant B cells with a marker profile close to that of CLL, such as CD5^+^ immature and naïve B cells. These considerations highlight the need for better ways of characterising NMBCs and differentiating them from CLL. Nevertheless, the NMBC population identified in this study still had important biological and clinical associations.

Second, although robust associations between NMBCs and multiple clinical endpoints were demonstrated and internally validated, additional validation using a separate cohort is required for further development of NMBC as a potential biomarker. This was not possible using existing flow cytometry MRD data from the RIAltO trial as the 4-colour technique used does not allow the simultaneous measurement of the seven markers that are required to reliably identify NMBCs. However, the recent advent of multiparametric flow cytometry now offers the opportunity to validate these findings in other CLL trials.

Third, in the rapidly evolving treatment landscape of CLL, our study was limited to patients treated with CIT, a modality that has largely been superseded by BTK and BCL-2 inhibitors. Nonetheless, the independence of the observed prognostic effects from a specific CIT regimen or the addition of idelalisib suggests potential relevance in the context of newer therapies, though further investigations are needed to confirm this. Finally, our cohort included only one patient with a 17p deletion, thereby limiting the applicability of our findings to this high-risk subgroup.

In conclusion, the present study provides the first demonstration that longitudinal evaluation of NMBCs, measured as a proportion of circulating mononuclear cells, is biologically relevant and prognostically important in CLL and contributes information beyond that provided by established prognostic factors including MRD. In particular, our findings raise the possibility that re-emergence of NMBCs after treatment reflects clearance of CLL from secondary lymphoid organs, thereby complementing the role of MRD as a marker of bone marrow clearance. This, in turn, sets the scene for additional studies to explore the prognostic value of circulating NMBCs combined with MRD in a range of different therapy settings.

## Supplementary Information

Below is the link to the electronic supplementary material.

Supplementary Material 1

## Data Availability

Datasets used for this study are available from the corresponding author upon reasonable request.
