# Supplementary material for: Re-emergence of circulating non-malignant B cells as a prognostic biomarker in chronic lymphocytic leukaemia
Source: Sci Rep. 2025 Sep 26;15:33072. doi: 10.1038/s41598-025-16558-5 (PMC12475484; doi:10.1038/s41598-025-16558-5)
Supplement: Supplementary file 1 — Supplementary Material 1 [file 41598_2025_16558_MOESM1_ESM.docx]

**Supplemental Information**

**RIAltO trial**

RIAltO (EudraCT 2011-000919-22) was an open-labelled, multicentre randomised controlled trial (RCT) that aimed to compare the second generation anti-CD20 monoclonal antibody ofatumumab with chlorambucil versus ofatumumab with bendamustine in patients with chronic lymphocytic leukaemia (CLL) who were considered not suitable for FCR (fludarabine, cyclophosphamide and rituximab) chemoimmunotherapy (CIT). Following the emergence of first-in-class phosphoinositide 3-kinase delta (PI3Kγ) inhibitor idelalisib for the treatment of CLL, an amendment was made to the trial protocol whereby recruited patients further underwent a double-blinded randomisation to receive CIT plus either additional placebo or idelalisib. Patients were treated with up to 6 cycles of ofatumumab plus bendamustine or up to 12 cycles of ofatumumab plus chlorambucil. Idelalisib or placebo was initiated alongside CIT and administered continuously until disease progression, intolerable toxicity or study discontinuation. 521 patients were recruited between December 2011 and April 2021 including 145 who were randomised to idelalisib vs placebo between September 2014 and March 2016 when the randomisation was closed due to safety concerns. The last patient was enrolled in April 2018 with follow-up completed in April 2021. The primary end point was progression-free survival (PFS). Secondary endpoints were response, response duration, overall survival and treatment toxicity. Disease response and progression were defined using the international workshop on Chronic Lymphocytic Leukaemia (iwCLL) criteria.

| **Covariate(s)** | **Level(s)** | **Univariable analysis** | |
| --- | --- | --- | --- |
|  |  | **Est (95% CI)** | **P-value** |
| Age (years) | <75 | Reference | |
|  | ≥75 | 1.276 (0.442, | 0.652 |
| ECOG performance score | 0-1 | Reference | |
|  | 2-3 | 1.096 (0.243, 4.938) | 0.905 |
| Sex | Female | Reference | |
|  | Male | 1.018 (0.282, 3.683) | 0.978 |
| Binet stage | A/B | Reference | |
|  | C | 0.728 (0.252, 2.103) | 0.558 |
| IgHV mutational status | Mutated | Reference | |
|  | Unmutated | 2.449 (0.661, 9.067) | 0.18 |
| 11p deletion | No | Reference | |
|  | Yes | 2.44 (0.3, 19.833) | 0.404 |
| 13q deletion | No | Reference | |
|  | Yes | 0.589 (0.191, 1.818) | 0.357 |
| Trisomy 12 | No | Reference | |
|  | Yes | 1.098 (0.296, 4.067) | 0.889 |
| Chemotherapy received | Chlorambucil | Reference | |
|  | Bendamustine | 1.355 (0.422, 4.355) | 0.61 |
| Idelalisib randomisation | Placebo | Reference | |
|  | Idelalisib | 0.563 (0.188, 1.688) | 0.305 |
| Post-treatment FBC: | | | |
| Haemoglobin (g/dL) | ≥12 | Reference | |
|  | <12 | 2.932 (0.997, 8.624) | 0.051 |
| Neutrophil count (x10^9/L) | ≥1.5 | Reference | |
|  | <1.5 | 2.951 (0.38, 22.951) | 0.301 |
| Platelet count (x10^9/L) | ≥150 | Reference | |
|  | <150 | 0.797 (0.275, 2.306) | 0.675 |
| Post-treatment immunoglobulin levels: | | | |
| Serum IgG (g/L)* | Continuous | 1.065 (0.9, 1.259) | 0.466 |
| Serum IgA (g/L)* | Continuous | 0.719 (0.33, 1.569) | 0.407 |
| Serum IgM (g/L)* | Continuous | 1.095 (0.816, 1.471) | 0.545 |
| Post-treatment MRD | <0.1% | Reference | |
|  | ≥0.1% | 1.078 (0.269, 4.328) | 0.916 |
| NMP clusters | Continuous | 0.93 (0.851, 1.016) | 0.109 |

**Table S1:** **Univariable analysis for overall survival following completion of chemoimmunotherapy**, **with estimated effect (Est), 95% confidence interval (CI) and statistical significance for all potential covariates**. ECOG: Eastern Cooperative Oncology Group, NMP: non-malignant phenotype. *samples with no available information were excluded.


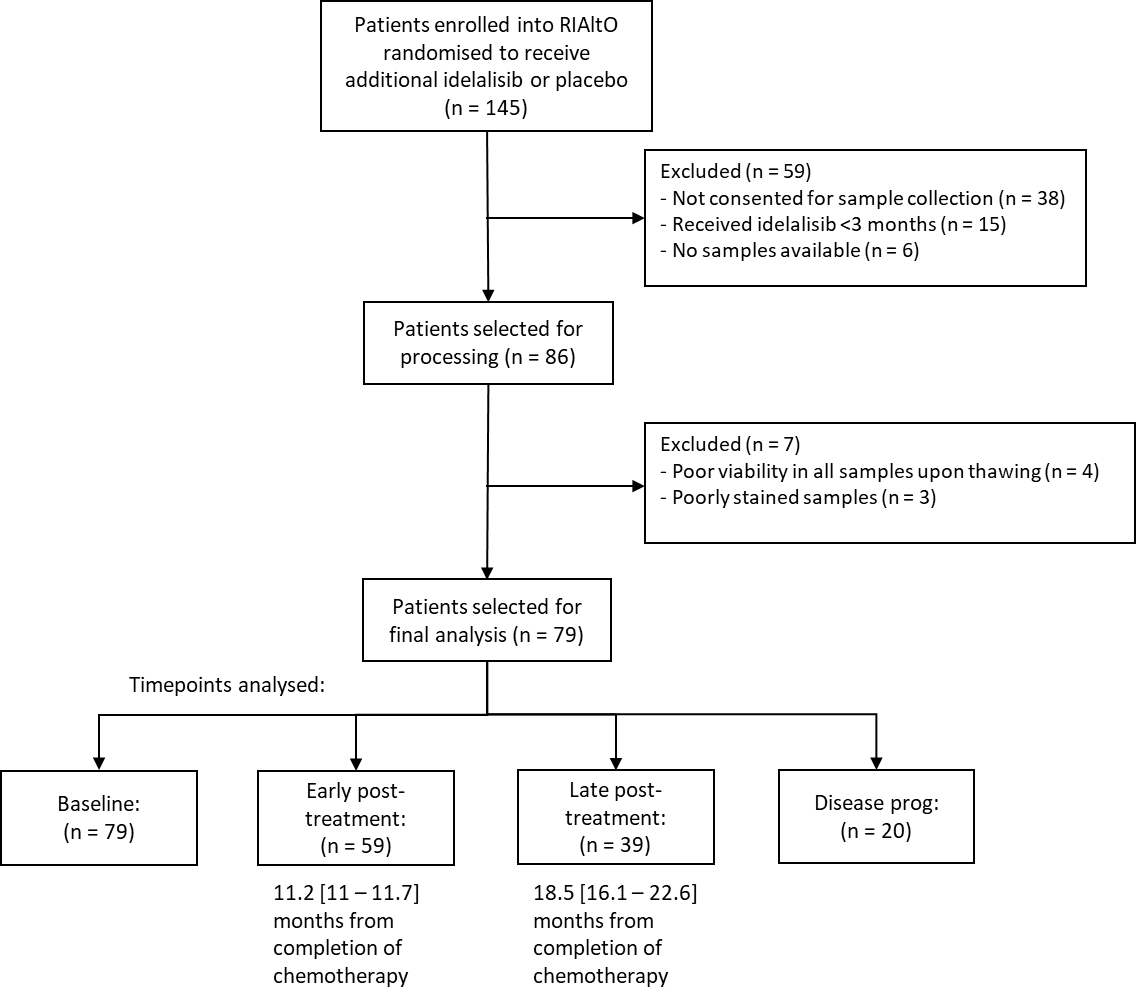


**Figure S1:** CONSORT diagram of RIAltO trial samples included in the analysis


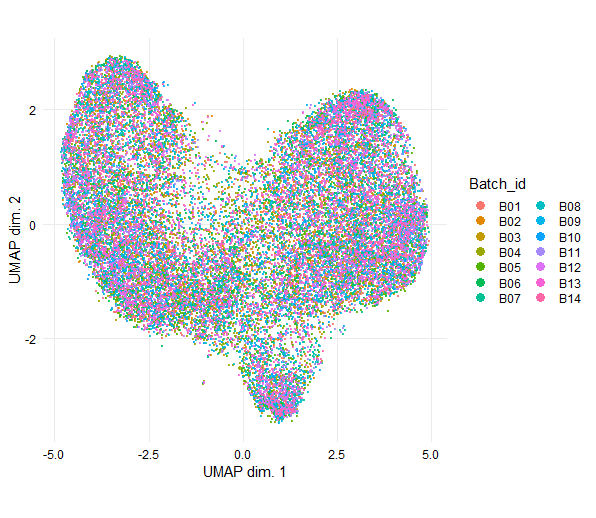

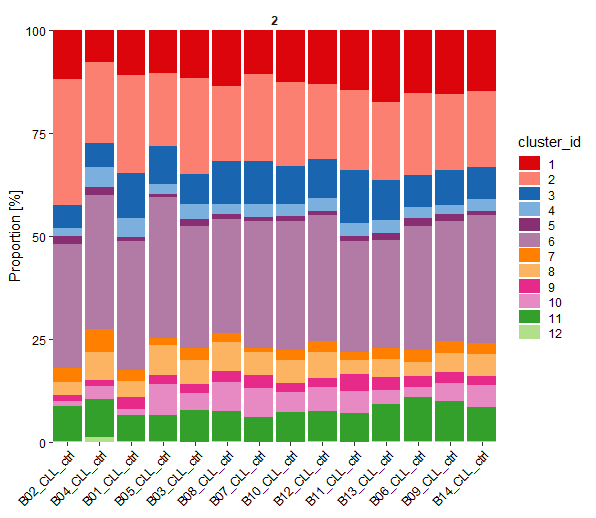


**Figure S2:** t-SNE analysis of CD19^+^ B cells from biological replicates processed in each batch, revealing a consistent global expression pattern across different batches (top). FlowSOM cluster proportions in biological replicates from each batch, showing similar relative frequencies of each cluster between batches (bottom).


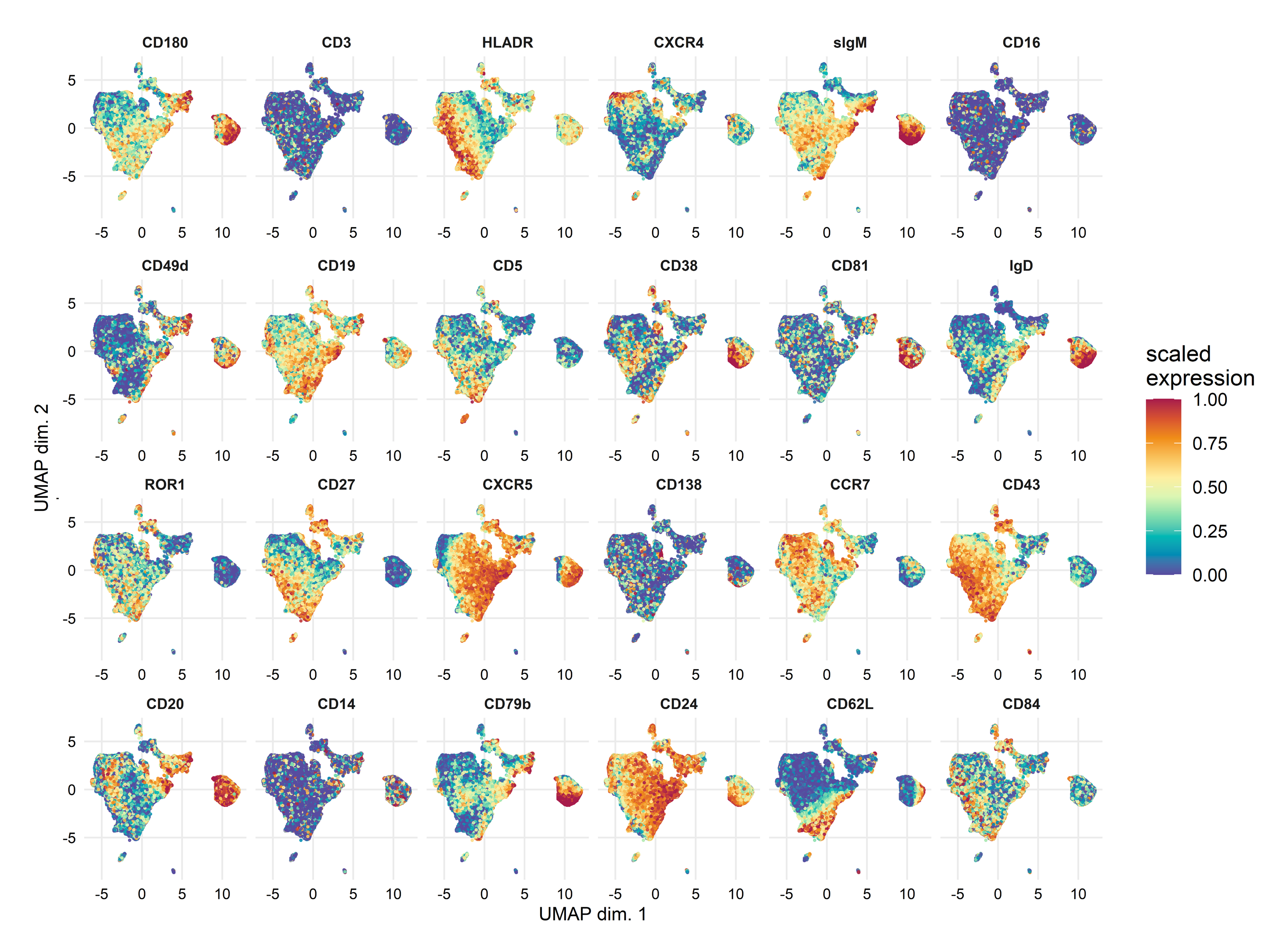


**Figure S3:** UMAPs showing expression profile of all analysed markers


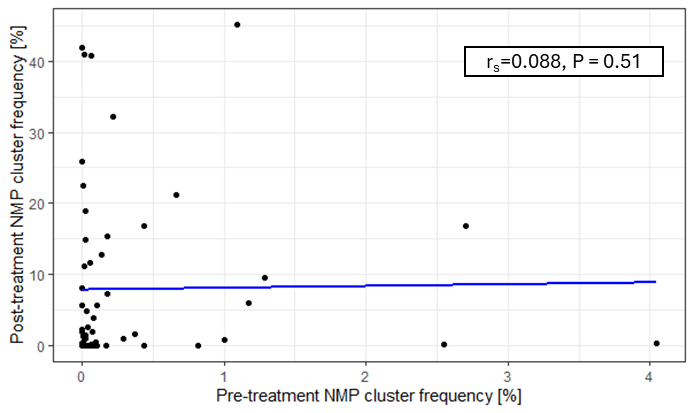


**Figure S4:** Scatter plot and linear regression analysis comparing total NMP cluster frequency between paired pre- and early post-treatment samples.


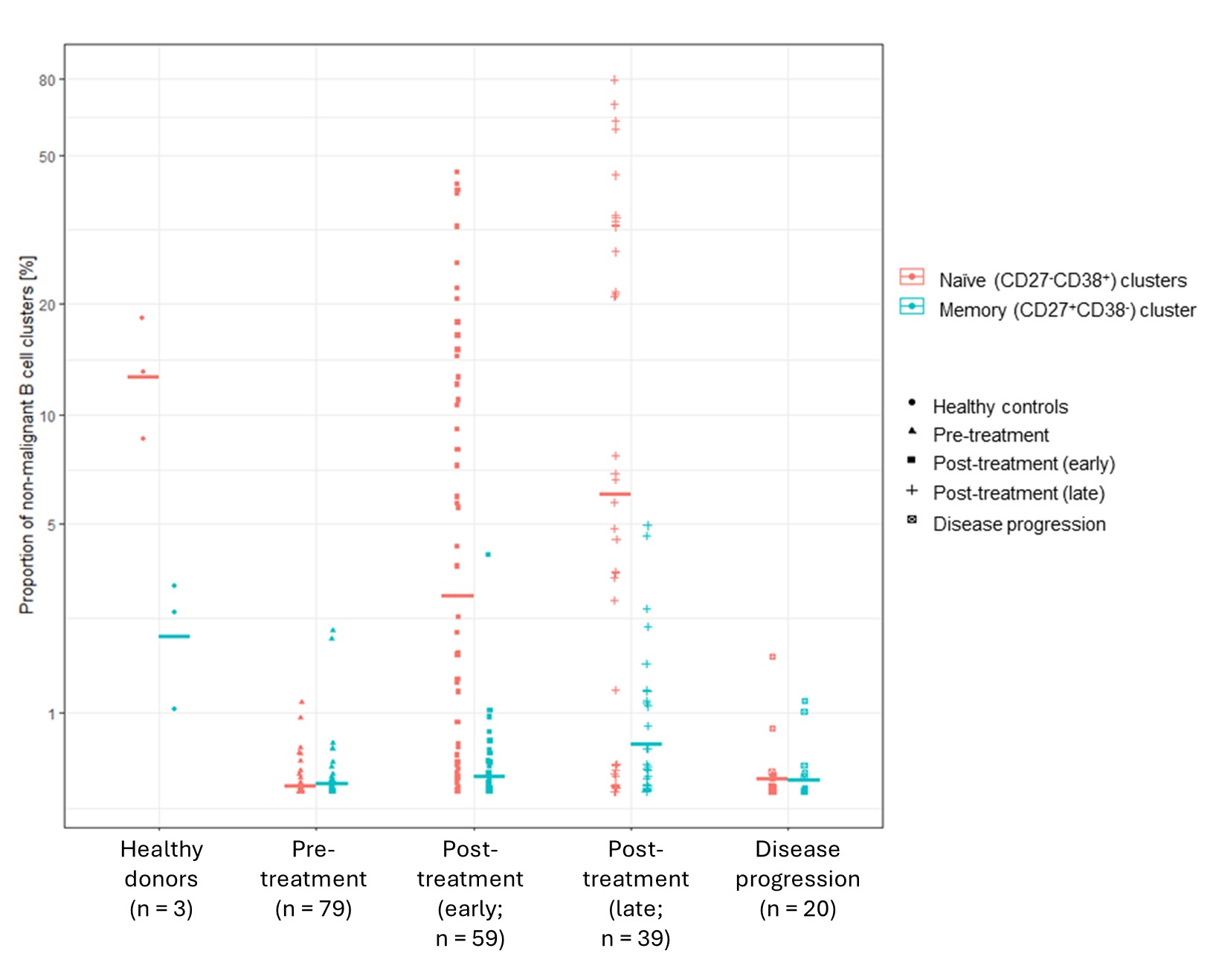
**Figure S5:** Proportion of naïve (CD27^-^CD38^+^) and memory (CD27^+^CD38^-^) NMP cluster(s) in healthy controls (HC) and patients with CLL at pre-treatment and post-treatment timepoints. Log10 transformation was applied to the y-axis to aid visualisation.


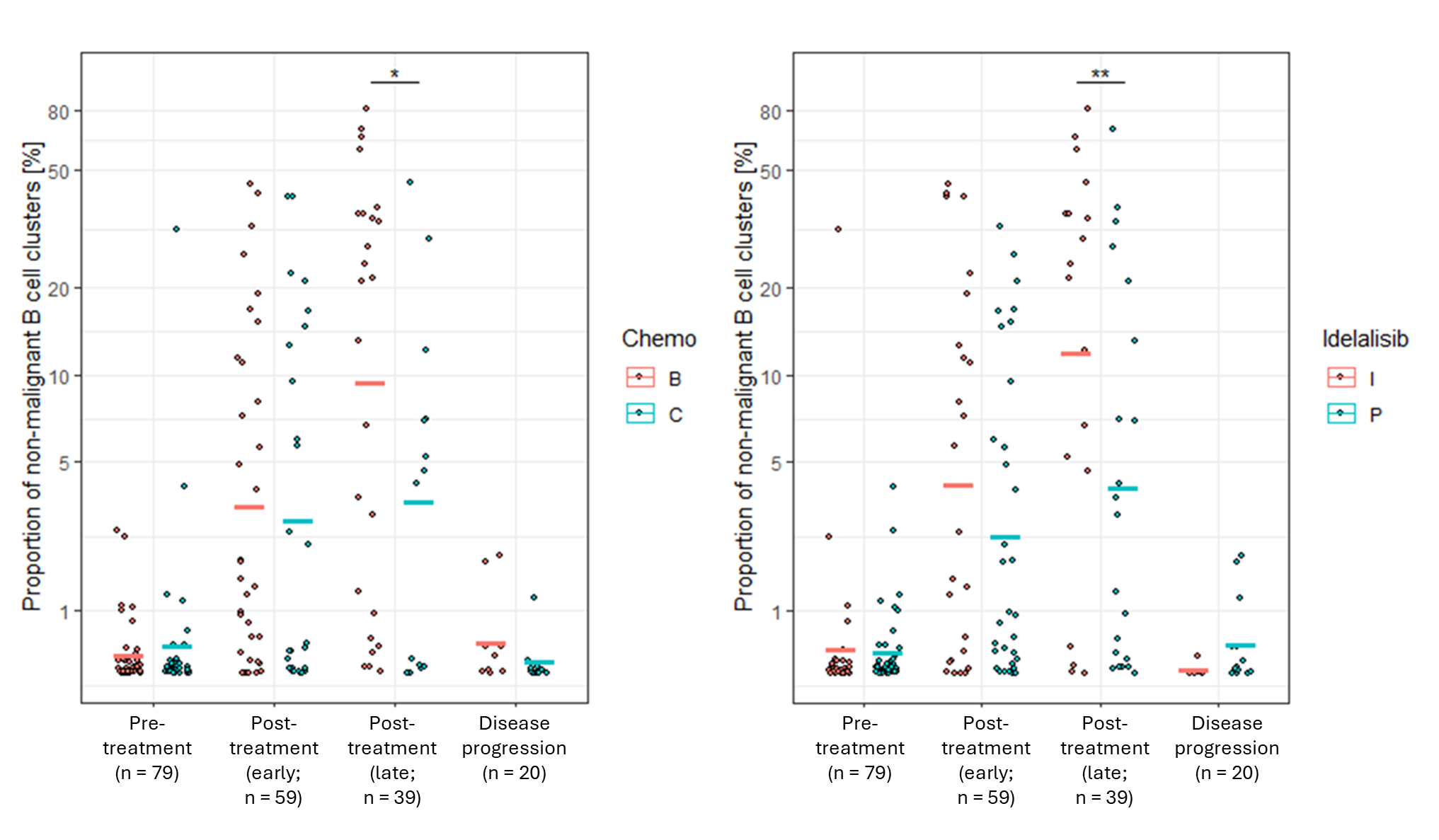


**Figure S6:** Proportion of total NMP clusters in patients with CLL at pre-treatment and post-treatment timepoints, split by chemotherapy allocation (left) and allocation to idelalisib vs placebo (right). B: bendamustine; C: chlorambucil; I: idelalisib; P: placebo. Log10 transformation was applied to the y-axis to aid visualisation


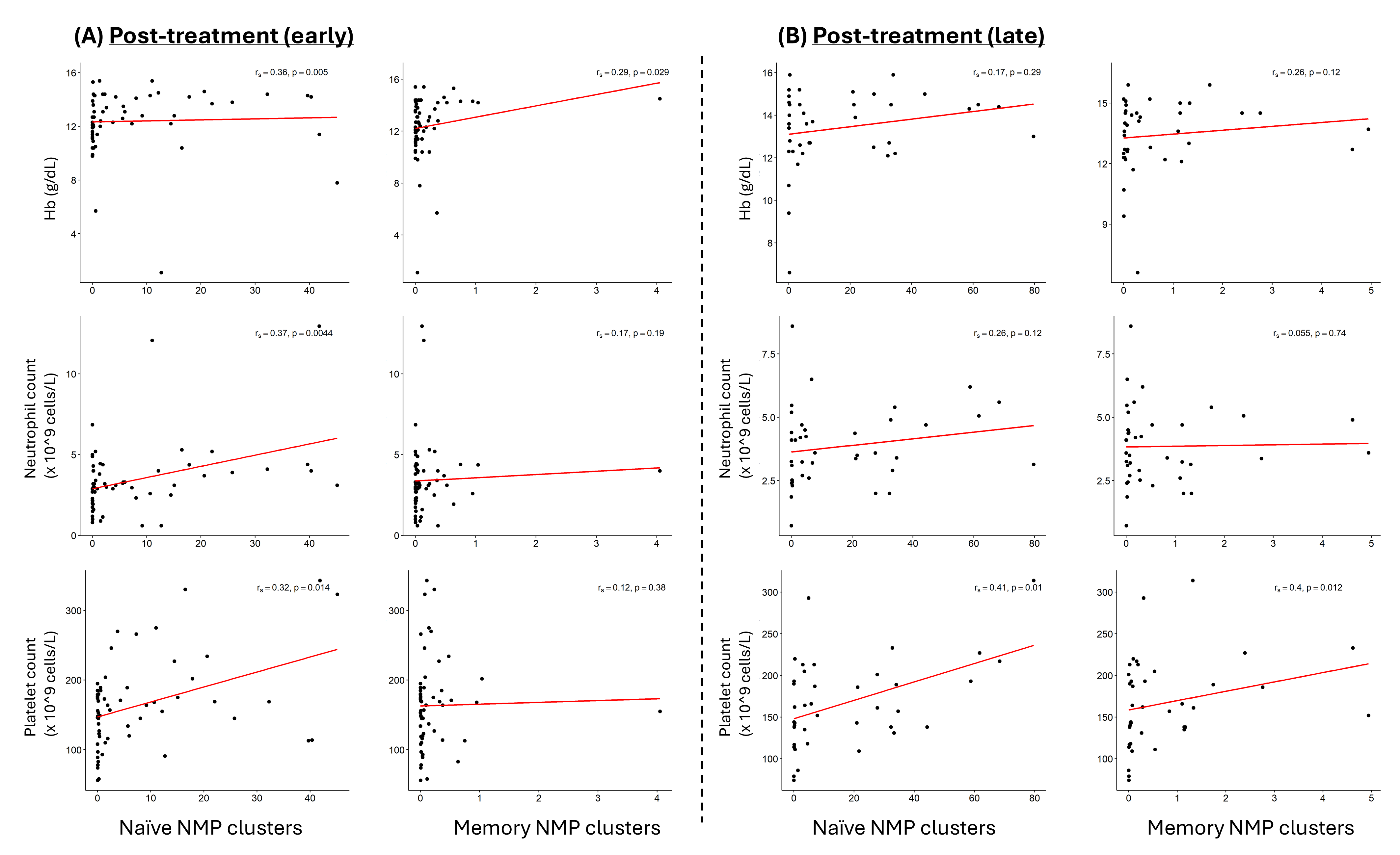


**Figure S7:** Scatter plots and linear regression analyses comparing naïve and memory NMP cluster frequencies with the recovery of blood counts following treatment. NMP: non-malignant phenotype.


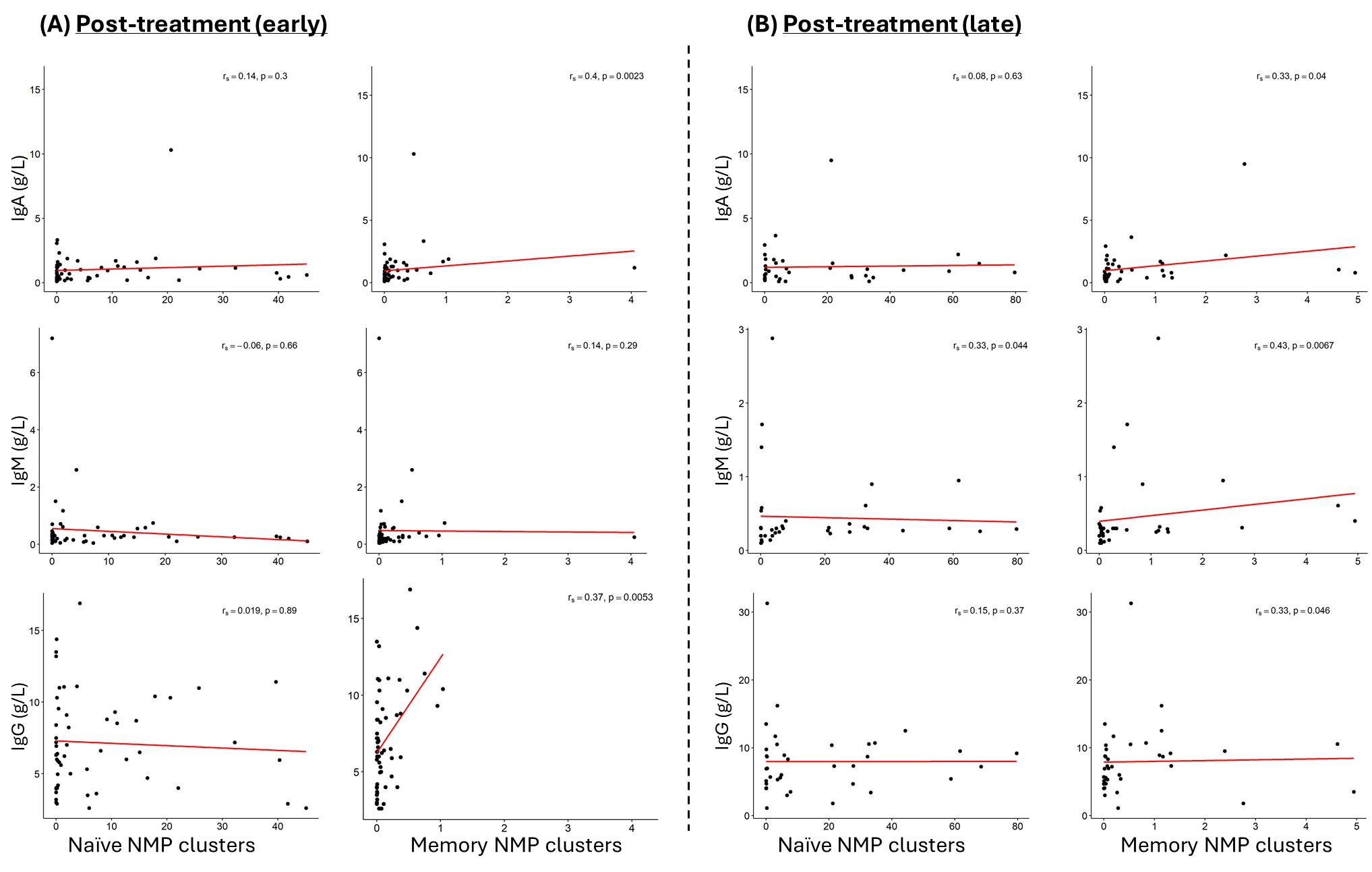


**Figure S8:** Scatter plots and linear regression analyses comparing naïve and memory NMP cluster frequencies with the recovery of immunoglobulin levels following treatment. NMP: non-malignant phenotype. At the early post-treatment timepoint, one patient was excluded due to missing serum immunoglobulin data, while an additional patient had available data for IgM and IgA but lacked information on IgG levels. One patient was excluded at the late post-treatment timepoint for the same reason.


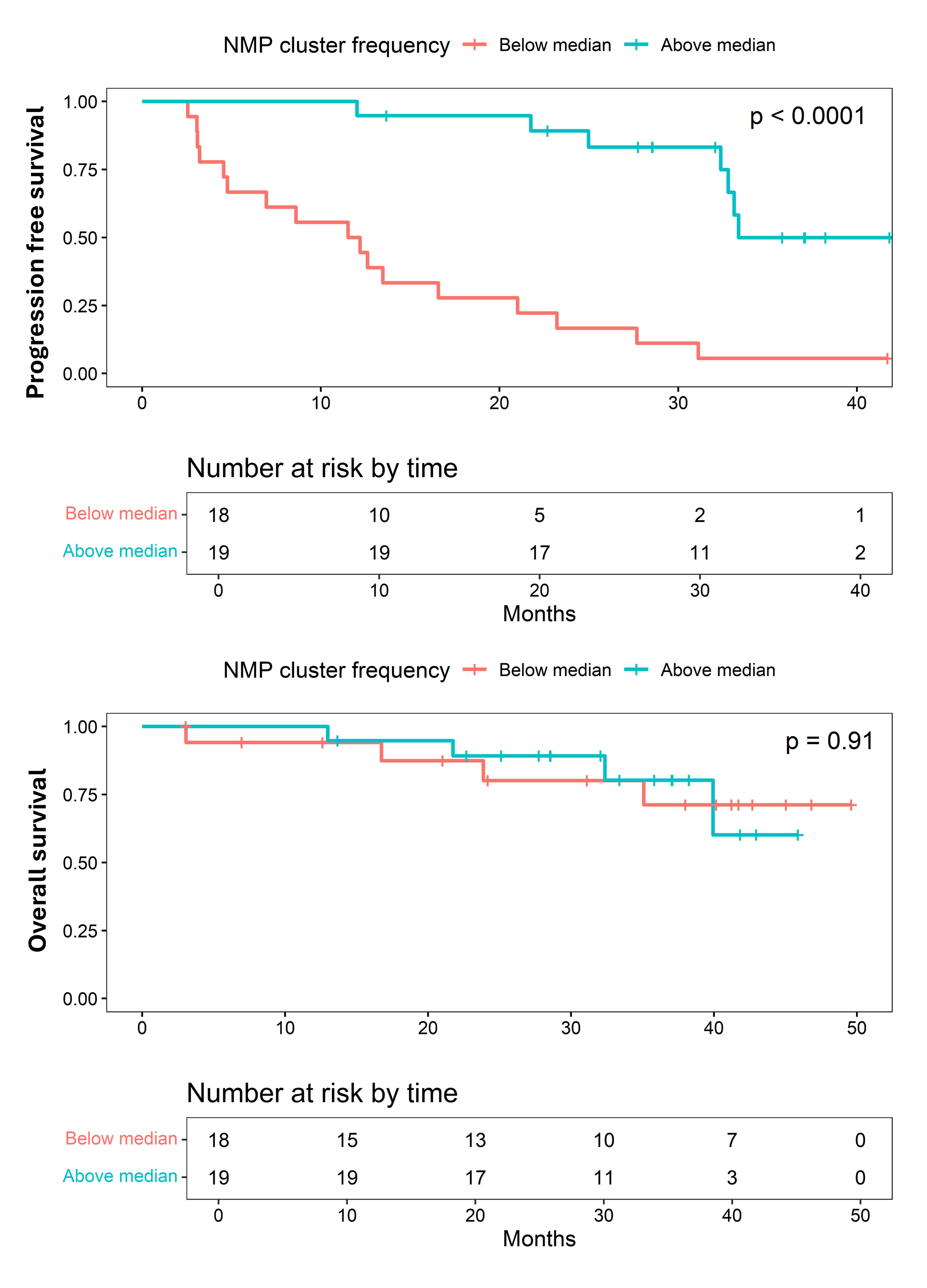


**Figure S9:** Kaplan-Meier (K-M) plot and log-rank test of progression free survival (top) and overall survival (bottom) comparing patients with FlowSOM-defined NMP clusters above or below the median value (7%) at the late post-CIT timepoint.


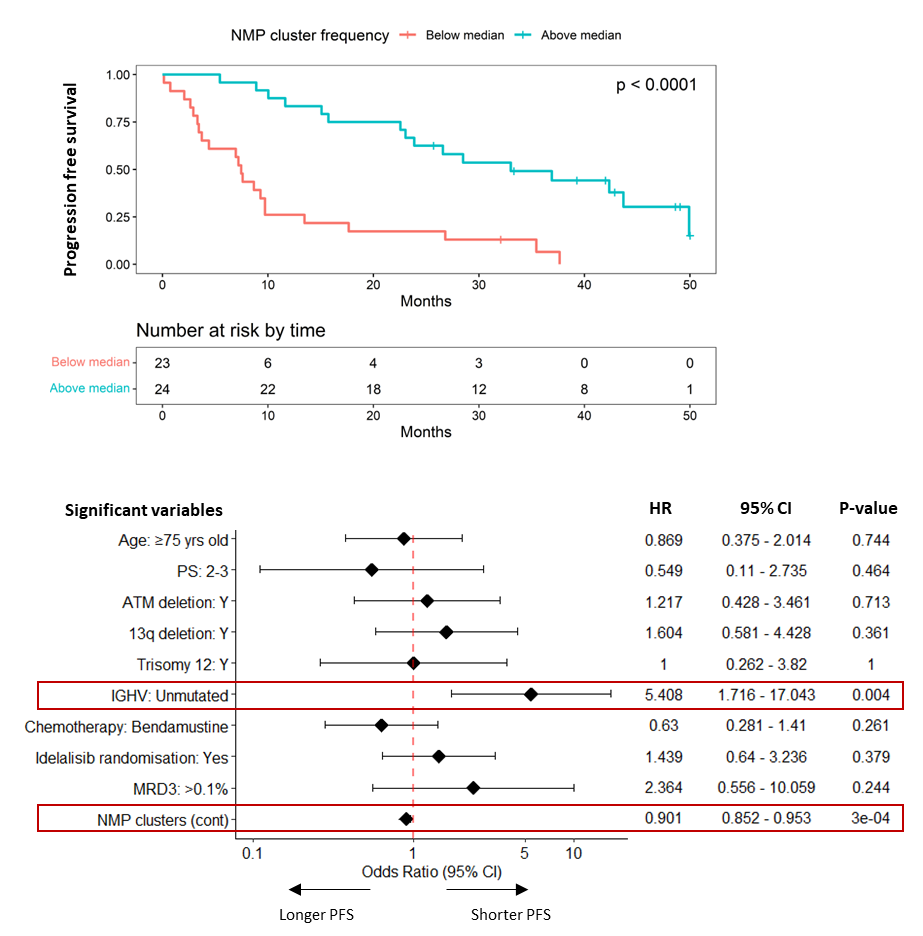


**Figure S10:** Analysis focused on samples with at least 1,000 total cells analysed (sensitivity of a least 0.001%). Kaplan-Meier (K-M) plot and log-rank test of progression free survival (PFS) comparing patients with FlowSOM-defined NMP clusters above or below the median value (1.95%) at the early post-CIT timepoint (top). Multivariable Cox proportional hazard analysis assessing the association between PFS and baseline characteristics, treatment allocation, post-CIT MRD status and NMP cluster size at the early post-CIT timepoint (bottom).


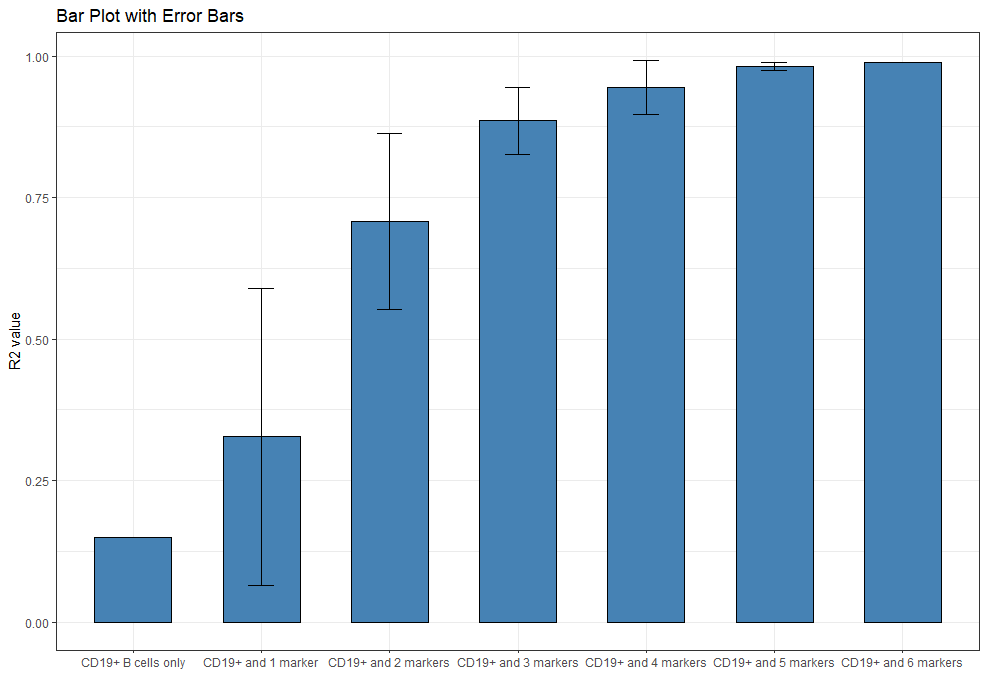


Spearman’s correlation (r_s_)

Lineage-defining markers* only

1 NMBC-defining marker

2 NMBC-defining markers

3 NMBC-defining markers

4 NMBC-defining markers

5 NMBC-defining markers

6 NMBC-defining markers

+ lineage-defining markers*

**Figure S11:** Spearman’s correlation co-efficient between NMBC frequency detected propectively using one or more NMP-defining markers and those detected using the unsupervised FlowSOM clustering approach. Spearman’s correlation was assessed for all possible combinations of the six NMBC-defining markers, including: six combinations with one marker, 15 combinations with two markers, 20 combinations with three markers, 15 combinations with four markers, 6 combinations with five markers, and one combination using all six markers. For groups with multiple combinations, the median correlation with interquartile range (IQR) is shown. For groups with only a single combination, the absolute correlation value is displayed. *Lineage defining markers include CD45, CD19, CD3.
